# MoEPH: an adaptive fusion-based LLM for predicting phage-host interactions in health informatics

**DOI:** 10.3389/fmicb.2025.1634705

**Published:** 2025-09-18

**Authors:** Qian Chen, Zihang Zhao, Min Li, Wenchen Song, Minfeng Xiao, Min Fang

**Affiliations:** ^1^School of Artificial Intelligence and National Engineering Laboratory for Big Data System Computing Technology, Shenzhen University, Shenzhen, Guangdong, China; ^2^School of Computer Science and Technology, The University of Hong Kong, Hong Kong, Hong Kong SAR, China; ^3^BGI-Shenzhen, Shenzhen, China; ^4^University of Chinese Academy of Sciences, Beijing, China; ^5^Education Center of Experiments and Innovations, Harbin Institute of Technology, Shenzhen, Guangdong, China

**Keywords:** trustworthy phage-host prediction, interpretable, robustness, transformer-based protein embeddings, mixture of experts (MoE)

## Abstract

Phage-host interaction prediction plays a crucial role in the development of phage therapy, particularly in combating antimicrobial resistance (AMR). Current in silico models often suffer from limited generalizability and low interpretability. To address these gaps, we introduce MoEPH (Mixture-of-Experts for Phage-Host prediction), a novel framework that integrates transformer-based protein embeddings (ProtBERT and ProT5) with domain-specific statistical descriptors. Our model dynamically combines features using a gated fusion mechanism, ensuring robust and adaptive prediction. We evaluate MoEPH on three publicly available phage-host interaction databases: Dataset 1 (101 host strains, 129 phages), Dataset 2 (38 host strains, 176 phages), and Dataset 3 (combined). Experimental results demonstrate that MoEPH outperforms existing methods, achieving an accuracy of 99.6% on balanced datasets and a 31% improvement on highly imbalanced data. The model provides a transparent, dynamic and knowledge-driven fusion solution for phage-host prediction, contributing to more effective phage therapy recommendations. Future work will focus on incorporating structural protein features and exploring alternative neural backbones for further enhancement.

## 1 Introduction

Antimicrobial resistance (AMR) has emerged as a global health crisis and a “silent pandemic,” threatening effective infection treatment worldwide. Without intervention, AMR is projected to cause up to 10 million deaths annually by 2050 (Walsh et al., 2023), with millions of lives already impacted each year. This dire situation has reignited interest in alternative therapeutics (Murray et al., 2022). Bacteriophage (phage) therapy—the use of viruses that specifically infect bacteria—has re-emerged as a promising strategy to combat drug-resistant infections. Phages can lyse antibiotic-resistant bacteria with high specificity, offering a potential lifeline where antibiotics fail. In this context, developing accurate phage—host identification methods is crucial to actualize phage therapy against AMR. Our work lies at the intersection of phage therapy and artificial intelligence, aiming to advance trustworthy medical AI to address this challenge. Moreover, increasing biological data resources facilitate this goal; for instance, we leverage a large phage–host interaction dataset from BGI-Shenzhen,^1^ which provides extensive phage genomic and host information to support model training and evaluation.

Existing *in silico* phage–host prediction methods, however, face significant limitations that hinder clinical utility. Early approaches rely on genomic sequence similarity or alignment-based heuristics, such as BLAST hits or CRISPR-spacer matches. Alignment-free statistical methods [e.g., WIsH (Galiez et al., 2017) and RaFAH (Coutinho et al., 2021)] predict hosts based on k-mer composition or protein content, but often fail on distantly related phages and have limited accuracy. Later methods introduced machine learning on genomic features: for example, logistic matrix factorization models (Leite et al., 2018) and network-based frameworks like VirHostMatcher-Net (Wang et al., 2020) integrated multiple genomic similarity measures to improve predictions. These offered moderate performance gains yet still struggle with generalizability and robustness, especially for novel phages or under-represented hosts. Deep learning has also been applied—notably PredPHI (Li et al., 2020), a CNN-based tool utilizing phage and host protein features—achieving higher accuracy than classical approaches. However, such deep models act as black boxes with limited interpretability, and their improvements in accuracy remain modest. Even knowledge-integrated approaches encounter challenges: a recent knowledge graph model KGVHI (Pan et al., 2024) combines multiple data types (genomic, proteomic, and host metadata) to predict microbe–host pairs and shows excellent performance on benchmark datasets, yet it requires comprehensive prior knowledge and is not specialized for bacteriophage therapy scenarios. In summary, existing methods tend to overfit to training data and imbalanced distributions, often over-predicting dominant hosts while missing rare interactions. They lack the adaptability to generalize to new phage or host species and provide little biological insight into predictions. Such opacity and instability undermine user trust, which is critical for AI deployment in healthcare. Thus, improvements in generalization, robustness, and explainability are essential before phage–host prediction models can be used confidently in clinical practice.

Meanwhile, large pre-trained models have brought transformative advances to bioinformatics. In the protein biology domain, protein language models (pLMs) like ProtBERT and ProT5 (Elnaggar et al., 2021) leverage Transformer architectures to learn rich protein sequence representations. These models capture structural motifs and achieve state-of-the-art performance in diverse biological tasks. Treating protein sequences as a “language of life,” such models offer superior feature learning for tasks like phage–host prediction. Similarly, in biomedical NLP, large domain-specific language models [e.g., PubMedBERT (Gu et al., 2021)] have demonstrated that pre-training on in-domain data yields powerful representations for downstream tasks. However, current pLM-based phage–host prediction approaches are often used only as static feature extractors, lacking dynamic adaptation or integration of domain knowledge. Recent studies have begun to endow language models with biological knowledge or multi-modal data (Chen et al., 2024), but a cohesive framework that combines pre-trained embeddings with adaptive, knowledge-driven fusion for phage–host prediction remains absent.

To address these gaps, we propose MoEPH, a Mixture-of-Experts framework designed to improve both performance and transparency for trustworthy phage–host prediction. MoEPH employs multiple expert subnetworks specializing in different feature modalities, with a gating network dynamically selecting experts for each input (Shazeer et al., 2017). This architecture enables adaptive, context-specific learning, effectively capturing both genomic composition signals and high-level protein patterns. By synergistically integrating pre-trained protein embeddings with interpretable statistical features, MoEPH achieves not only superior predictive performance but also enhanced explainability. Specifically, our model maintains robust accuracy even on highly imbalanced and novel data, mitigating the overfitting to dominant hosts that plagues prior methods. It also provides interpretability through per-sample expert weight analysis, offering biological insight into which features drive a given prediction. These qualities align with key pillars of trustworthy AI—reliability, explainability, and adaptability (Aljohani et al., 2025)—making MoEPH particularly suitable for sensitive applications like phage therapy recommendation. In summary, our main contributions are an innovative multi-expert fusion strategy tailored for phage–host prediction, a comprehensive evaluation demonstrating state-of-the-art performance across multiple datasets, and an analysis showing improved generalization and interpretability compared to existing models.

In summary, our main contributions are:

- **Trusted phage-host prediction framework:** We develop MoEPH, combining pre-trained embeddings with dynamic expert selection to enhance robustness and interpretability in clinical AI applications targeting AMR.
- **Enhanced robustness on imbalanced data:** MoEPH achieves stable performance even under highly imbalanced conditions, avoiding overfitting and reliably predicting under-represented bacteria.
- **Interpretability via expert weight visualization:** The gating network output offers interpretable insights into model decisions, supporting clinician trust and biological discovery.
- **Generalization across datasets and novel pairs:** Extensive evaluation shows that MoEPH generalizes well to external datasets and unseen phage-host pairs, maintaining reliability as scientific knowledge evolves.

## 2 Definition and materials

In pursuit of trustworthy AI, this chapter details the feature design and data preparation that form the foundation for MoEPH, with a particular emphasis on explainability and robustness. Specifically, we integrate knowledge-driven statistical descriptors with context-rich transformer-based protein embeddings to balance interpretability and predictive performance. Fundamental features such as amino acid composition (AAC), atomic composition (AC), and molecular weight (MW) provide human-understandable sequence characteristics that complement the deep sequence representations from pretrained models (e.g., ProtBERT, ProT5). By unifying these two classes of features within a Mixture-of-Experts (MoE) framework, our model can more effectively capture protein complexity in an adaptive manner. The synergy between interpretable statistical features and advanced LLM-derived embeddings thus serves as the basis for a more explainable and resilient predictive system. In this section, we describe the designed statistical features and the datasets utilized, which together underpin the trustworthy modeling approach of MoEPH.

### 2.1 Definition of statistical features

We incorporate three interpretable, domain-knowledge-driven statistical descriptors to complement deep embeddings:

(1) **Amino acid composition (AAC):** Measures the frequency of each amino acid *A*_*i*_ in sequence *S*:


$$\text{AA}{\text{C}}_{i}=\frac{n_{i}}{L},\;i=1,\ldots ,M,$$

where *n*_*i*_ is the count of *A*_*i*_ and *L* is the sequence length. AAC provides coarse but robust information for classification tasks.

(2) **Atomic composition (AC):** Represents the proportion of each element *E*_*j*_ (e.g., C, H, N, O, S) in a protein:


$$\text{A}{\text{C}}_{j}=\frac{a_{j}}{\sum_{k=1}^{E}a_{k}},\;j=1,\ldots ,E,$$

where *a*_*j*_ is the number of atoms of *E*_*j*_ in *S*. AC captures the protein's fundamental chemical makeup.

(3) **Molecular weight (MW):** Summarizes compositional information into a physicochemical metric:


$$\text{MW}\left(S\right)=\overset{M}{\underset{i=1}{\sum }}n_{i}\cdot M\left(A_{i}\right),$$

where *M*(*A*_*i*_) denotes the molecular weight of amino acid *A*_*i*_. MW can discriminate proteins based on mass profiles.

These statistical features enhance model explainability by providing intuitive biochemical insights into protein sequences.

### 2.2 Transformer-based LLMs protein representations

We leverage ProtBERT and ProT5, two pre-trained protein LLMs, to generate context-rich sequence embeddings.

(1) ProtBERT: ProtBERT is a BERT-based protein language model that employs a bidirectional Transformer encoder with a masked language modeling (MLM) objective [20]. During pre-training, a subset *M* = {*m*_1_, …, *m*_*K*_} of positions in the input sequence *S* is randomly masked, and the model learns to predict the masked residues using context. The MLM loss is defined as:


(1) $$\begin{array}{l} L_{\text{MLM}}\left(\theta \right)=-\underset{m\in M}{\sum }\log P\left(s_{m}|S_{\backslash m};\theta \right), \end{array}$$

where *S*_\*m*_ denotes *S* with the residue at position *m* replaced by a mask token. ProtBERT stacks multiple Transformer layers to iteratively refine the sequence representation:


(2) $$\begin{array}{l} H^{\left(l\right)}=\text{TransformerLayer}H^{\left(l-1\right)},\;l=1,2,\ldots ,L, \end{array}$$

with *H*^(0)^ being the input token embeddings. By leveraging contextual cues, ProtBERT learns both local motifs and long-range dependencies, yielding robust sequence features for downstream tasks.

(2) ProT5: ProT5 is a T5-based encoder–decoder model that frames protein modeling tasks in a sequence-to-sequence format. It can be pre-trained to reconstruct corrupted sequences, predict functional or structural annotations, or even generate novel protein sequences. Given an input sequence *S* and a target sequence *Y* = {*y*_1_, …, *y*_*T*_}, ProT5 models the conditional probability:


(3) $$\begin{array}{l} P\left(Y|S;\theta \right)=\overset{T}{\underset{t=1}{\prod }}P\left(y_{t}|y_{<t},S;\theta \right). \end{array}$$

It learns by minimizing the negative log-likelihood:


(4) $$\begin{array}{l} L_{\text{T5}}\left(\theta \right)=-\overset{T}{\underset{t=1}{\sum }}\log P\left(y_{t}|y_{<t},S;\theta \right). \end{array}$$

The encoder first produces a hidden representation:


(5) $$\begin{array}{l} H_{\text{enc}}=\text{Encoder}\left(S\right). \end{array}$$

The decoder then uses *H*_enc_ (together with previously generated tokens *y*_<*t*_) to predict the next token *y*_*t*_:


(6) $$\begin{array}{l} H_{\text{dec},t}=\text{Decoder}H_{\text{enc}},y_{<t},\;t=1,2,\ldots ,T. \end{array}$$

Through this process, ProT5 captures rich long-range dependencies and excels in generative and multi-task settings. Training on diverse objectives endows ProT5 with a broad latent space of protein sequences, complementing ProtBERT's embeddings.

### 2.3 Dataset of the immersion experiment

The datasets originate from a BGI research project titled *Research on Artificial Phage Model Construction Based on Deep Generative Adversarial Network Learning* (see text footnote ^1^), providing a reliable empirical foundation for our prediction tasks. This study is part of an ongoing initiative at BGI-Shenzhen to explore phage-host prediction under real-world data constraints. The equipment is based on the NVIDIA A100 cloud platform. To evaluate the model under multi-source conditions, we utilize two distinct datasets and a third integrated dataset. To evaluate the model under multi-source conditions, we utilize two distinct datasets and a third integrated dataset.

**Figure 1:**
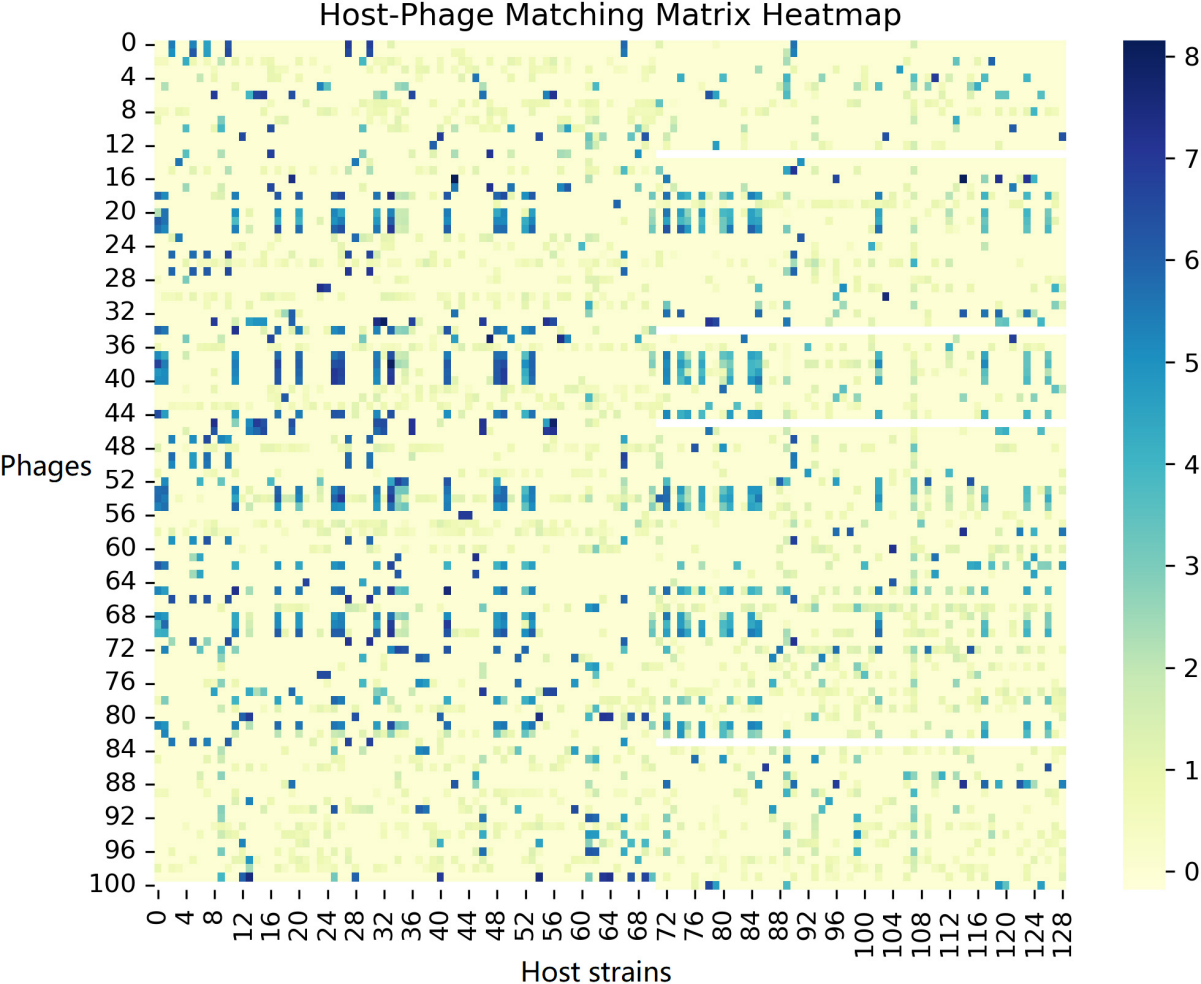
Host-phage matching matrix.

To provide a clearer picture of the dataset composition, we include a heatmap of the host-phage interaction matrix (Figure 1) in the data description. This figure offers an overview of which phages infect which host strains in Dataset 1, highlighting the data's structure before any modeling.

**Dataset 1:** This dataset includes 101 host bacterial strains and 129 phages, collected under well-defined conditions, ensuring clear information on species composition and environmental context.

**Dataset 2:** This dataset comprises 38 host strains and 176 phages. Compared to Dataset 1, its sampling conditions (e.g., environment and strain selection) differ, yielding distinct phage–host interaction patterns.

**Dataset 3:** Dataset 3 combines Dataset 1 and Dataset 2 to enable performance assessment under more diverse conditions, providing a robust test of the model's adaptability to heterogeneous data sources in real-world settings.

(1) *Immersion experiment and labeling strategy:* The phage–host interactions were measured via immersion experiments, where phages were exposed to host cultures and infection outcomes recorded in a host–phage matrix (Figure 1). We binarized these outcomes by labeling interactions with infection values above 1.5 as 1 (significant infection) and below 1.5 as 0 (no significant infection), ensuring a clear separation between positive and negative samples.

(2) *Sequence information and feature extraction:* For each host and phage, we obtained the protein amino acid sequence and extracted features including traditional descriptors (AAC, AC, MW) and LLM-based embeddings (ProtBERT, ProT5), yielding a rich feature set for learning.

In summary, these curated multi-source datasets and comprehensive feature sets provide a solid foundation to assess the model's generalization and robustness. Their diversity underscores the importance of MoEPH's dynamic fusion mechanism for reliable predictions across heterogeneous conditions.

## 3 Proposed framework: MoEPH

In this section, we present the architecture of the proposed **M**ixture-**o**f-**E**xperts model for **P**hage **H**ost prediction (MoEPH). The MoEPH framework is designed to leverage transformer-based protein representations and an ensemble of expert sub-models to address the complex task of phage-host prediction. Mixture-of-Experts (MoE) architectures have also been used in recent large language models to achieve greater scalability by dividing the model's knowledge among specialized sub-networks; we adopt a similar principle here to effectively handle the heterogeneity of phage-host data. By decomposing the prediction task among multiple expert networks and using an adaptive gating mechanism to fuse their outputs, MoEPH can capture diverse patterns in the data. This design enhances the model's robustness and flexibility, as each expert can specialize in certain features or sub-distributions of the input, while the gating network dynamically selects and combines expert contributions appropriate for each phage query. In what follows, we detail the overall model framework, the structure of the Mixture-of-Experts layer, and the training and inference procedures.

### 3.1 MoEPH model framework

**Figure 2:**
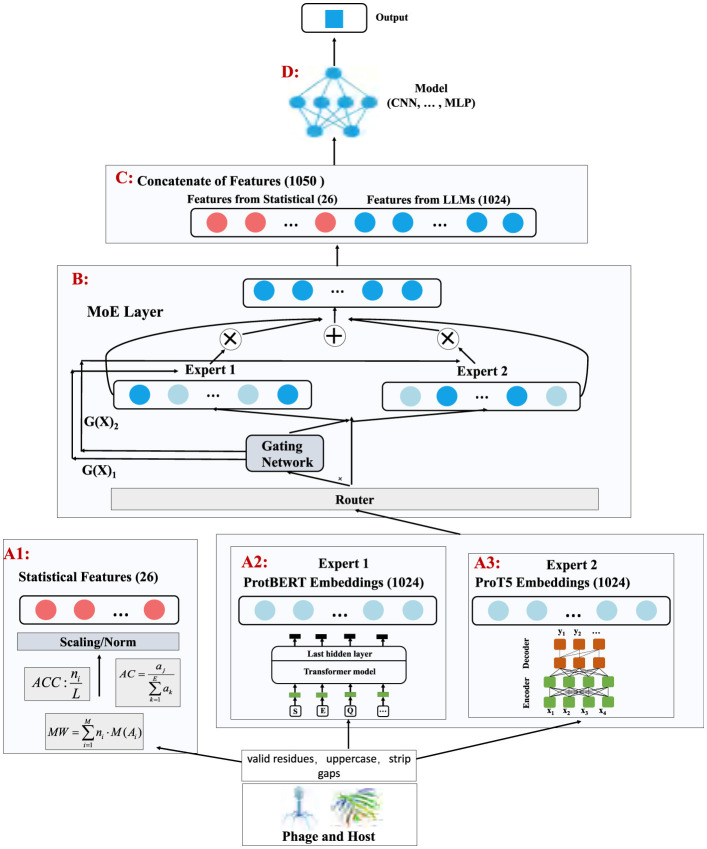
Flowchart of MoEPH. This figure illustrates the model's main components, including statistical feature extraction (A1), two Transformer-based LLM feature extraction modules (A2, A3), the MoE layer (B) for adaptive expert weighting, feature concatenation (C), and the final prediction model (D).

The MoEPH model proposed in this study is designed to integrate multi-source features both statistical descriptors and deep sequence embeddings extracted by large language models (LLMs) and to adaptively weight these features through a Mixture-of-Experts (MoE) mechanism. By doing so, the model produces more robust and expressive representations for subsequent classification tasks. Figure 2 provides an overview of the framework.

In **A1 (Statistical feature extraction)**, we derive fundamental statistical descriptors such as amino acid composition (AAC), atomic composition (AC), and molecular weight (MW) from each protein sequence, denoted as ${\text{X}}_{\text{stats}}\in {\mathbb{R}}^{N\times 26}$ in the example illustration. These features reflect basic physicochemical properties of the protein and serve as an initial numeric reference for subsequent integration.

Concurrently, in **A2** and **A3 (LLM feature extraction)**, we employ two pre-trained protein language models: ProtBERT and ProT5 to capture deep semantic representations of the sequences, resulting in ${\text{X}}_{\text{ProtBERT}}\in {\mathbb{R}}^{N\times 1024}$ and ${\text{X}}_{\text{ProT5}}\in {\mathbb{R}}^{N\times 1024}$. These high-dimensional embeddings encode contextual, local structural, and functional information within the sequences, thereby enriching the predictive power for phage-host interaction tasks.

Next, these three kinds of features are fed into **B: the MoE layer**, which consists of a **Router** and two expert modules (Expert 1 and Expert 2). A gating network processes the statistical features **X**_stats_ to generate weights α, dictating how **X**_ProtBERT_ and **X**_ProT5_ should be combined. This adaptive weighting ensures that the model can dynamically select the most discriminative or best-matched expert features for each sample under varying data distributions and feature patterns. In addition, the resulting weighting coefficients can provide interpretability regarding the relative importance of each LLM for different types of samples.

As depicted in **C (Concatenate of features)**, the fused features obtained from the MoE layer are concatenated with the statistical features, producing ${\text{X}}_{\text{combined}}\in {\mathbb{R}}^{N\times 1050}$ (illustrative dimensionality). This consolidated vector representation fully integrates both the “protein statistical attributes” and the “LLM-based deep features.”

Finally, **D (Model)** processes **X**_combined_ using a customizable prediction network (e.g., a CNN, MLP, or RNN) to output the phage-host interaction results. In this study, we employ a CNN classifier to systematically examine how the fused features improve performance (e.g., accuracy, F1-score, AUC) under a fixed network structure.

In summary, MoEPH incorporates statistical features from traditional analyses and advanced embeddings from ProtBERT/ProT5, then uses an adaptive MoE layer to effectively and interpretably combine multi-source information. This integrated solution provides a robust yet flexible approach to phage-host interaction prediction.

### 3.2 MoE layer of the MoEPH model

In protein-related prediction tasks, relying solely on a single pre-trained model (e.g., ProtBERT or ProT5) often fails to fully capture the diverse sequence patterns and structural information inherent in biological data. To address this limitation, we introduce a Mixture-of-Experts (MoE) mechanism into our model. By combining multiple pre-trained experts and adaptively assigning their importance based on sample-specific statistical attributes, the MoE layer flexibly merges various feature advantages, thereby enhancing predictive performance and overall generalization (Shazeer et al., 2017; Pearce and Zhang, 2021). The entire fusion procedure is summarized in Algorithm 1.

**Algorithm 1:**
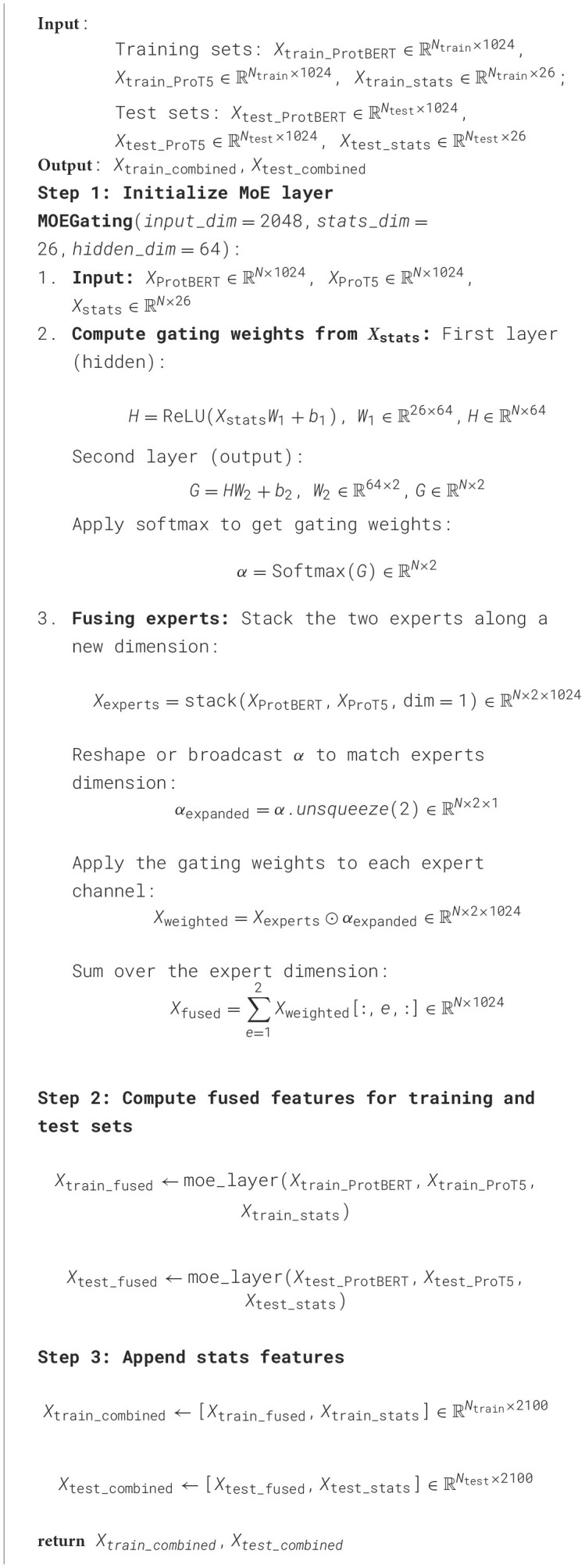
Mixture-of-Experts (MoE) layer feature fusion in MoEPH.

### 3.3 MoE layer structure and gating mechanism

In our Mixture-of-Experts (MoE) layer, a *gating network* dynamically computes sample-specific weights to fuse the outputs of two expert models (ProtBERT and ProT5). Formally, for each sample *i*, let $X_{\text{ProtBERT},i}\in {\mathbb{R}}^{d}$ and $X_{\text{ProT5},i}\in {\mathbb{R}}^{d}$ denote the pre-trained feature vectors from ProtBERT and ProT5 (with *d* = 1024 in our implementation). Each sample also has an associated statistical feature vector $X_{\text{stats},i}\in {\mathbb{R}}^{s}$ (with *s* = 26 descriptors such as physicochemical properties and sequence composition). The gating function *g*(·) is realized as a two-layer feed-forward network that transforms the statistical features into a pair of importance weights (α_*i*, 1_, α_*i*, 2_) for the two experts. Concretely, the gating network first applies a fully-connected layer to *X*_stats, *i*_ (shared across samples) to produce a hidden representation, then a second fully-connected layer produces two logit scores which are normalized by a softmax activation:


(7) $$\begin{array}{ll} H & =\text{ReLU}X_{\text{stats}}W_{1}+b_{1},\;H\in {\mathbb{R}}^{N\times 64}, \end{array}$$

(8) $$\begin{array}{ll} G & =HW_{2}+b_{2},\;G\in {\mathbb{R}}^{N\times 2}, \end{array}$$

(9) $$\begin{array}{ll} \alpha & =\text{softmax}\left(G\right),\;\alpha \in {\mathbb{R}}^{N\times 2}, \end{array}$$

where $W_{1}\in {\mathbb{R}}^{s\times 64},b_{1}\in {\mathbb{R}}^{64},W_{2}\in {\mathbb{R}}^{64\times 2},b_{2}\in {\mathbb{R}}^{2}$ are trainable parameters. The ReLU activation in Equation 7 introduces nonlinearity into the gating function, and the softmax in Equation 9 ensures each sample's two gating coefficients (α_*i*, 1_, α_*i*, 2_) form a probability distribution (i.e., α_*i*, 1_, α_*i*, 2_≥0 and α_*i*, 1_+α_*i*, 2_ = 1 for each *i*). Importantly, this gating is *dynamic and sample-specific*: for each sample *i*, the statistical attribute vector *X*_stats, *i*_ yields its own gating weights α_*i*, 1_ and α_*i*, 2_. This design allows the model to adaptively decide how much to rely on each expert's features based on the characteristics of that sample (rather than using fixed static fusion weights).

Using the gating weights α_*i*, 1_ and α_*i*, 2_, the MoE layer modulates and fuses the expert outputs for each sample. Let α_*i*_ = [α_*i*, 1_, α_*i*, 2_] be the weight vector for sample *i*. We obtain the *fused* feature for sample *i* by an element-wise weighted sum of the two expert feature vectors:


(10) $$\begin{array}{l} X_{\text{fused},i}=\alpha_{i,1}X_{\text{ProtBERT},i}+\alpha_{i,2}X_{\text{ProT5},i},\;X_{\text{fused},i}\in {\mathbb{R}}^{d}. \end{array}$$

In other words, the ProtBERT embedding is scaled by α_*i*, 1_ and the ProT5 embedding by α_*i*, 2_, and then they are added together to produce a single fused representation for sample *i*. Stacking these results for all *N* samples yields the fused feature matrix:


(11) $$X_{\text{fused}}=\;\alpha_{1}⊙X_{\text{ProtBERT}}+alpha_{2}⊙X_{\text{ProT5}}\;\in \;{\mathbb{R}}^{N\times d}\;,$$

where $\alpha_{:,1},\alpha_{:,2}\in {\mathbb{R}}^{N\times 1}$ denote the two columns of α (broadcasted across the *d*-dimensional feature vectors), and ⊙ denotes element-wise (Hadamard) product. Through Equations 10–12, the gating weights effectively *modulate* the contribution of each expert: if α_*i*, 1_≫α_*i*, 2_ for a given sample, the fused representation *X*_fused, *i*_ will be dominated by ProtBERT's features, whereas if α_*i*, 2_ is larger, ProT5's features are emphasized. This adaptive fusion flexibly leverages the strengths of both experts, allowing the model to favor the expert that is more informative for each particular sample's attributes.

After obtaining the fused LLM-based features *X*_fused_, we integrate them with the original statistical features. Specifically, we concatenate each sample's fused vector with its statistical descriptor vector to form the final combined feature:


(12) $$\begin{array}{l} X_{\text{combined}}=\left[X_{\text{fused}},X_{\text{stats}}\right]\in {\mathbb{R}}^{N\times \left(d+s\right)}. \end{array}$$

In our implementation *d* = 1024 and *s* = 26, so $X_{\text{combined}}\in {\mathbb{R}}^{N\times 1050}$. This concatenation preserves the original handcrafted features alongside the fused deep features, ensuring that downstream classifiers receive a comprehensive feature set. The entire gated fusion procedure is summarized in Algorithm 2.

**Algorithm 2:**
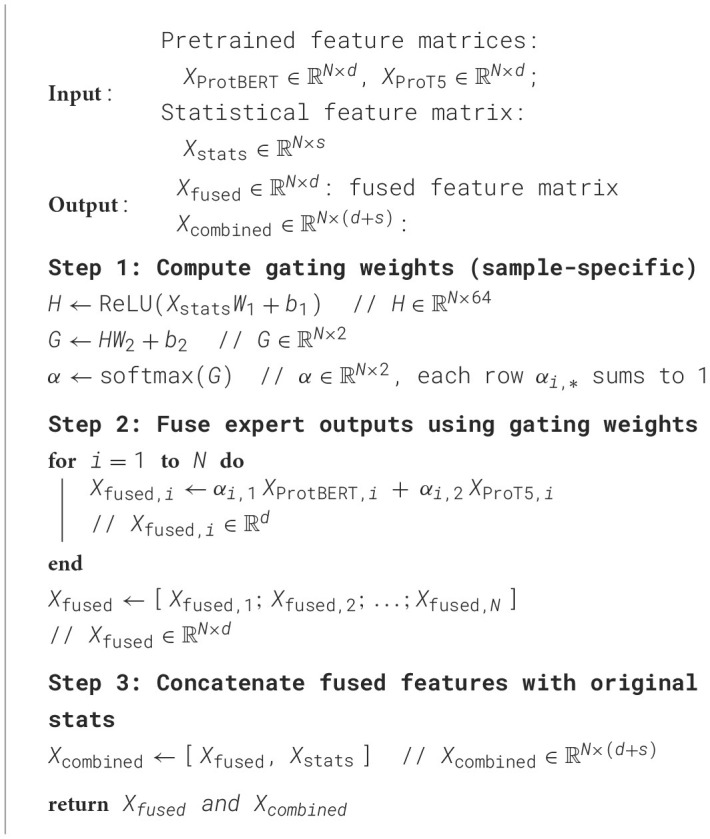
Pseudocode of MoE gating and expert fusion.

### 3.4 Model training and prediction

After extracting and preprocessing the features, we employ a convolutional neural network (CNN) as the classification backbone for phage-host interaction prediction, integrating the MoE module for feature fusion throughout the training process. While we fix this CNN architecture for consistency, our main objective is to demonstrate how the proposed MoEPH framework leverages multi-source embeddings to boost predictive performance, the advantage would extend similarly to other model architectures. The overall training and evaluation pipeline is summarized in Algorithm 3.

**Algorithm 3:**
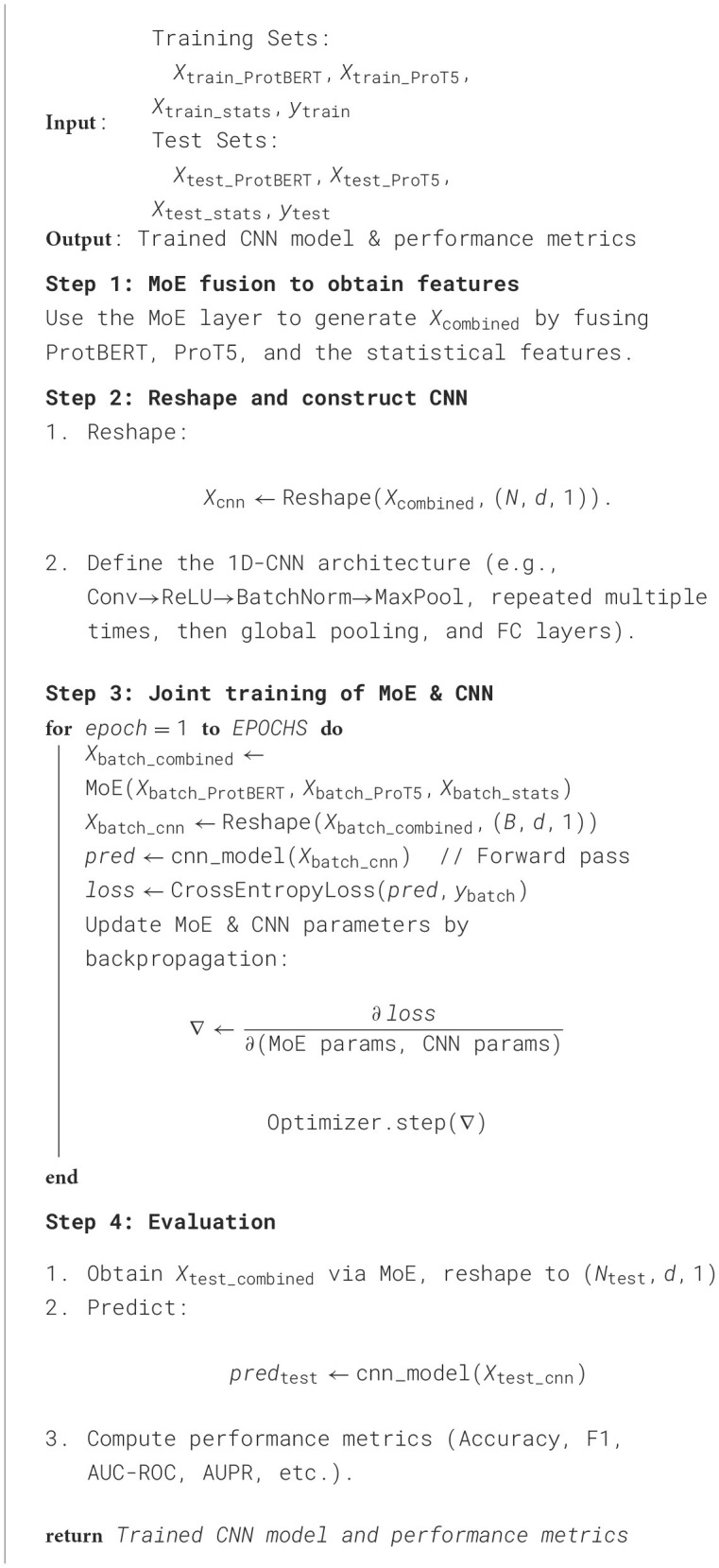
Model training and evaluation (CNN with MoE fusion).

#### 3.4.1 Network architecture

Let *X*∈ℝ^*N*×*d*^ be the combined feature matrix obtained via the MoE fusion layer, where *N* is the number of samples and *d* is the dimensionality of the fused features (including both Transformer-based protein embeddings and statistical descriptors). Each sample $x_{i}\in {\mathbb{R}}^{d}$ is reshaped into a (*d*, 1) array, which is then fed into a series of 1D-CNN layers. Specifically, these layers consist of:

- One-dimensional convolutional layers (e.g., kernel_size = 3, varying channel widths), each followed by a nonlinear activation function (ReLU), batch normalization, and max-pooling;
- Global pooling operation to reduce the final convolution outputs to a fixed-size feature vector;
- Fully connected (FC) layers that project the pooled feature vector into logits for the binary classification task (interaction vs. non-interaction).

Additional regularization elements (e.g., dropout) may be introduced in the FC layers to mitigate overfitting.

#### 3.4.2 Loss function

We adopt the cross-entropy loss for binary classification:


(13) $$\begin{array}{l} L_{i}=-\left[y_{i}\log\left({\hat{p}}_{i}\right)+\left(1-y_{i}\right)\log\left(1-{\hat{p}}_{i}\right)\right], \end{array}$$

where *y*_*i*_∈{0, 1} is the true label for sample *i*, and ${\hat{p}}_{i}$ is the predicted probability that sample *i* is positive (i.e., displays phage-host interaction). Concretely, let *z*_*i*, 0_ and *z*_*i*, 1_ be the logits for the negative and positive classes, respectively, so that


(14) $$\begin{array}{l} {\hat{p}}_{i}=\frac{\exp\left(z_{i,1}\right)}{\exp\left(z_{i,0}\right)+\exp\left(z_{i,1}\right)}. \end{array}$$

Averaging over the entire training set of size *N* yields


(15) $$\begin{array}{l} L\left(\theta \right)=-\frac{1}{N}\overset{N}{\underset{i=1}{\sum }}\left[y_{i}\log\left({\hat{p}}_{i}\right)+\left(1-y_{i}\right)\log\left(1-{\hat{p}}_{i}\right)\right]. \end{array}$$

Minimizing *L*(θ) with respect to the model parameters θ steers the CNN toward more accurate probability estimates.

#### 3.4.3 Training procedure

We initialize the CNN parameters θ randomly and adopt the Adam optimizer with a selected learning rate (e.g., 1 × 10^−3^). Training proceeds in mini-batches over a fixed number of epochs. For each mini-batch:

1. We obtain the fused features *X*_batch_combined_ via the MoE layer.
2. Reshape them to (*B, d*, 1) for the 1D-CNN, where *B* is the mini-batch size.
3. Perform a forward pass through the CNN to compute logits and subsequently derive predicted probabilities.
4. Compute the cross-entropy loss using the predicted probabilities and the ground truth labels.
5. Backpropagate to compute gradients ∇_θ_*L*(θ) and update all relevant parameters (MoE and CNN) in one unified step.

This joint optimization ensures that the CNN learns discriminative features while the MoE layer continues to refine the expert-selection gating.

#### 3.4.4 Prediction

After convergence, we apply the trained model to the test set. Specifically, the MoE layer fuses the expert embeddings for each test sample, the fused feature vectors are reshaped for the CNN, and the final probabilities ${\hat{p}}_{j}$ are obtained via a Softmax layer. We use a threshold of 0.5 to determine the predicted class:


(16) $${\hat{y}}_{j}=\{\begin{array}{ll} 1, & \text{if }{\hat{p}}_{j}\geq 0.5,\\ 0, & \text{otherwise}. \end{array}$$

Standard classification metrics (accuracy, F1-score, AUC-ROC, AUPR, etc.) are then computed to evaluate predictive performance.

## 4 Experimental results

### 4.1 Data description and sampling strategies

This study evaluates the proposed model on three datasets (Dataset1, Dataset2, and a merged Dataset3 which combines the former two). Dataset 1 contains phage–host pairs collected under relatively consistent experimental conditions (with well-defined species compositions and environmental factors), whereas Dataset 2 comes from a more complex ecological background, yielding greater heterogeneity in phage–host interaction patterns. By merging these two sources into Dataset 3, we impose more stringent demands on the model's adaptability to heterogeneous, multi-source inputs. This strategy was chosen to preserve each dataset's unique characteristics and to assess whether the model generalizes across different sources. Had we merged the datasets from the beginning, any source-specific patterns or performance differences would be hidden. By first testing on each dataset individually, we can demonstrate MoEPH's robust performance under each condition, and then confirm its adaptability on the merged Dataset 3.

To further challenge the model's robustness, each dataset is examined under three class imbalance settings: the original imbalanced distribution (Raw), an Over-sampling variant, and an Under-sampling variant. In Over-sampling, instances from the minority class are replicated to balance the number of positive and negative samples (e.g., if *N*_*pos*_ and *N*_*neg*_ denote the counts of positive and negative samples with *N*_*pos*_<*N*_*neg*_, additional positive instances are randomly duplicated until *N*′*pos*≈*Nneg*). In Under-sampling, the opposite approach is applied: majority-class instances are randomly removed until *N*′*neg*≈*Npos*, thereby equalizing class counts. These three sampling methods simulate varying degrees of class imbalance encountered in real-world scenarios, enabling a comprehensive evaluation of the model's robustness across different data distributions.

#### 4.1.1 Sampling methods overview

**Figure 3:**
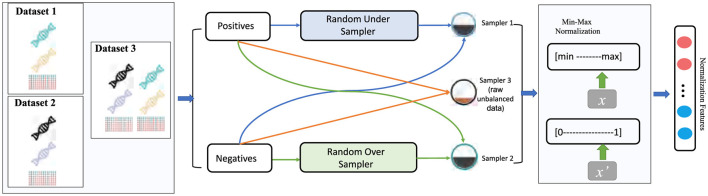
Data preprocessing pipeline for imbalanced datasets: under sampling, oversampling, and raw data.

To validate our model's performance under different class distributions and assess its generalization capability, we applied three sampling strategies to each dataset (as illustrated in Figure 3):

- **Raw (imbalanced data):** Directly using the original dataset while preserving its natural ratio of positive and negative samples, without any additional sampling.
- **Over-sampling:** Replicating instances from the minority class to balance the number of positive and negative samples. For instance, if *N*_*pos*_ and *N*_*neg*_ denote the number of positive and negative samples respectively and *N*_*pos*_<*N*_*neg*_, then over-sampling randomly duplicates some positive samples until $N_{pos}^{\prime }\approx N_{neg}$.
- **Under-sampling:** The opposite approach, which randomly removes part of the majority class to match the minority class size. If *N*_*pos*_<*N*_*neg*_, we randomly eliminate some negative samples so that $N_{neg}^{\prime }\approx N_{pos}$.

These three sampling methods simulate varying degrees of class imbalance encountered in real-world scenarios, offering a more comprehensive evaluation of the model's adaptability and generalization performance across different data distributions.

##### 4.1.1.1 Min-max normalization

**Figure 4:**
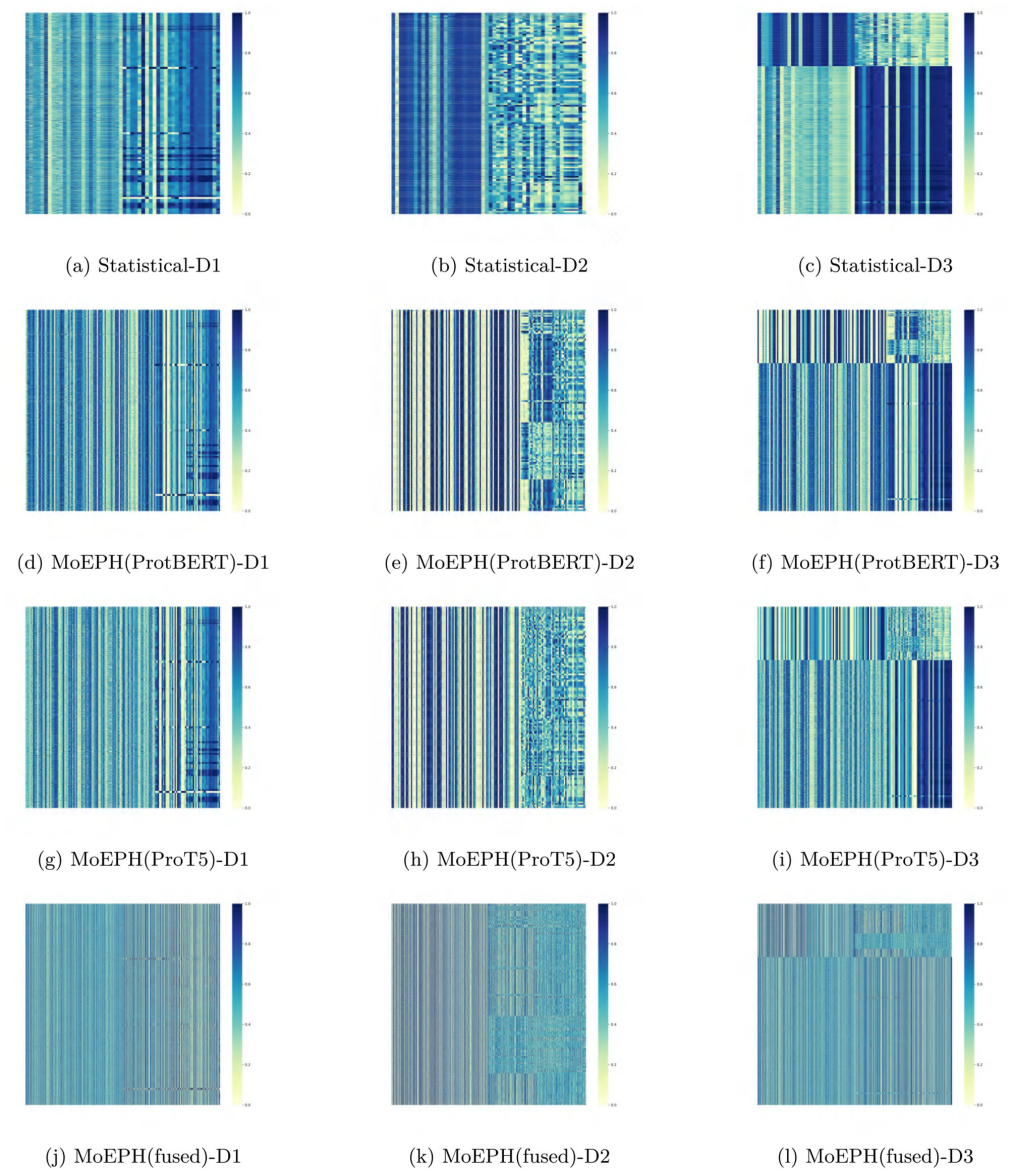
Heatmap of features in algorithms on different datasets. **(a)** Statistical-D1. **(b)** Statistical-D2. **(c)** Statistical-D3. **(d)** MoEPH(ProtBERT)-D1. **(e)** MoEPH(ProtBERT)-D2. **(f)** MoEPH(ProtBERT)-D3. **(g)** MoEPH(ProT5)-D1. **(h)** MoEPH(ProT5)-D2. **(i)** MoEPH(ProT5)-D3. **(j)** MoEPH(fused)-D1. **(k)** MoEPH(fused)-D2. **(l)** MoEPH(fused)-D3.

In addition to resampling, we apply a uniform preprocessing to all features. Min-Max Normalization: To alleviate discrepancies in feature value ranges, each feature is rescaled to [0, 1] via:


(17) $$\begin{array}{l} x^{\prime }=\frac{x-\min\left(x\right)}{\max\left(x\right)-\min\left(x\right)}, \end{array}$$

where *x* is an original feature value and *x*′ is its normalized counterpart. This normalization expedites model convergence and enhances stability, especially when combining features of different scales. Finally, to assess the Generality and Applicability of our approach, we design experiments from multiple perspectives:

- **Class distribution impact:** We compare model performance on naturally imbalanced data (Raw) versus balanced data (Over-sampled or Under-sampled) to gauge robustness to uneven class distributions.
- **Multi-source backgrounds:** By evaluating the algorithms on Dataset1, Dataset2, and the combined Dataset 3, we examine generalization under different biological settings and mixed conditions. This tests how well the model adapts to multi-source data variability, highlighting its robustness in a heterogeneous scenario.
- **Feature visualization:** Although the heatmaps (e.g., Figure 4) primarily reflect the distribution of final fused features rather than changes before sampling, contrasting the feature patterns generated by different datasets and algorithms still offers useful insights into what aspects the model focuses on and how those relate to potential biological interpretations.

### 4.2 Performance evaluation metrics

In this study, we adopt several widely recognized metrics to evaluate the predictive capability of our model under class-imbalanced conditions (Fawcett, 2006; Davis and Goadrich, 2006; Saito and Rehmsmeier, 2015; Huang and Ling, 2005). Below, we provide their mathematical definitions and briefly discuss their relevance in the context of phage–host interaction prediction.


(18) $$\begin{array}{l} \text{ACC}=\frac{TP+TN}{TP+FP+TN+FN} \end{array}$$

The proportion of all predictions that are correct.


(19) $$\begin{array}{l} \text{Prec}=\frac{TP}{TP+FP} \end{array}$$

The proportion of predicted positive cases that are truly positive.


(20) $$\begin{array}{l} \text{Spec}=\frac{TN}{TN+FP} \end{array}$$

The proportion of actual negative cases correctly identified (true negative rate).


(21) $$\begin{array}{l} F1=\frac{2\times \text{Precision}\times \text{Recall}}{\text{Precision}+\text{Recall}} \end{array}$$

The harmonic mean of precision and recall.


(22) $$\begin{array}{l} \text{AUC}=\int_{0}^{1}\text{TPR}\left(f\right)df \end{array}$$

The area under the ROC curve (TPR vs. FPR), summarizing performance across all thresholds.


(23) $$\begin{array}{l} \text{AUPR}=\int_{0}^{1}\text{Precision}\left(r\right)dr \end{array}$$

The area under the precision–recall curve, reflecting the precision–recall trade-off.

### 4.3 Feature representation analysis

Figure 4 provides a comparative visualization of the final feature representations produced by different methods across the three datasets. Each vertical column corresponds to a specific feature channel, and the color intensity indicates the magnitude of the feature values (with darker shades representing higher values and lighter shades indicating lower values). These heatmaps are derived from features output by our MoEPH model after the expert fusion layer, just before classification, under various configurations: using only statistical features, using ProtBERT embeddings, using ProT5 embeddings, and using the fused MoE approach (ProtBERT+ProT5). Compared to the traditional statistical baseline, the MoEPH-based models display more distinct block and stripe patterns, indicating that MoEPH captures finer-grained, context-dependent sequence features than conventional methods. Each subfigure corresponds to one method on one dataset (Figures 4a–l), allowing side-by-side comparison of how feature distributions vary by method and data complexity. The key observations are as follows:

#### 4.3.1 Statistical method vs. MoEPH-based features

Compared to the “Statistical Method” (see Figures 4a–c), the MoEPH variants (ProtBERT only, ProT5 only, and the fused approach) typically yield more distinct vertical stripes or block patterns in their heatmaps, with sharper gradients across various feature columns. Because the Statistical Method relies on basic statistical measurements, its derived feature vectors often exhibit relatively homogeneous distribution patterns. By contrast, the MoEPH approaches, having leveraged large pre-trained models, are better able to capture fine-grained, context-dependent sequence representations, leading to more pronounced differences among samples.

#### 4.3.2 Differences between ProtBERT and ProT5

As shown in Figures 4d–i, using ProtBERT versus ProT5 for feature extraction can produce noticeably different heatmap patterns in certain feature columns. In some dimensions, ProtBERT's response appears more “striped,” whereas ProT5 may display broader regions of lighter or darker shades. This indicates that the two pre-trained models have distinct sensitivities or biases when encoding the same sequence information. Such disparities become even more pronounced for Dataset 2 and Dataset 3, suggesting each model exhibits unique strengths in capturing sequence features under more complex biological conditions.

#### 4.3.3 Performance of the dated fusion [MoEPH (fused)]

Figures 4j–l present the heatmaps of features obtained by gating and fusing the ProtBERT and ProT5 experts. These images reveal that while certain ProtBERT-like or ProT5-like textures remain, some local regions appear smoothed out or amplified. This indicates that the gating mechanism is not merely an averaging step but rather a selective weighting process driven by statistical features and sequence context, yielding a more diversified feature distribution in complex data scenarios.

#### 4.3.4 Changes across datasets

A vertical comparison from Dataset 1 through Dataset 3 shows that as the dataset size and heterogeneity increase, the color patterns—blocks and stripes—also become more pronounced. For instance, in Dataset 3, which includes more varied samples, the color intensity for a given feature column may fluctuate more widely across different instances, suggesting that the large pre-trained models have learned broader sequence distinctions. Conversely, if a method is relatively insensitive to environmental variation, its heatmaps may exhibit similar patterns across datasets, implying limited adaptability in its learned feature space.

Overall, these visual observations suggest that MoEPH-based methods manifest more distinct block structures in their features and are better equipped than traditional statistical approaches to capture deeper sequence-level variations—an advantage that can underpin improved classification results in subsequent experiments.

### 4.4 Performance comparison of and analysis

**Figure 5:**
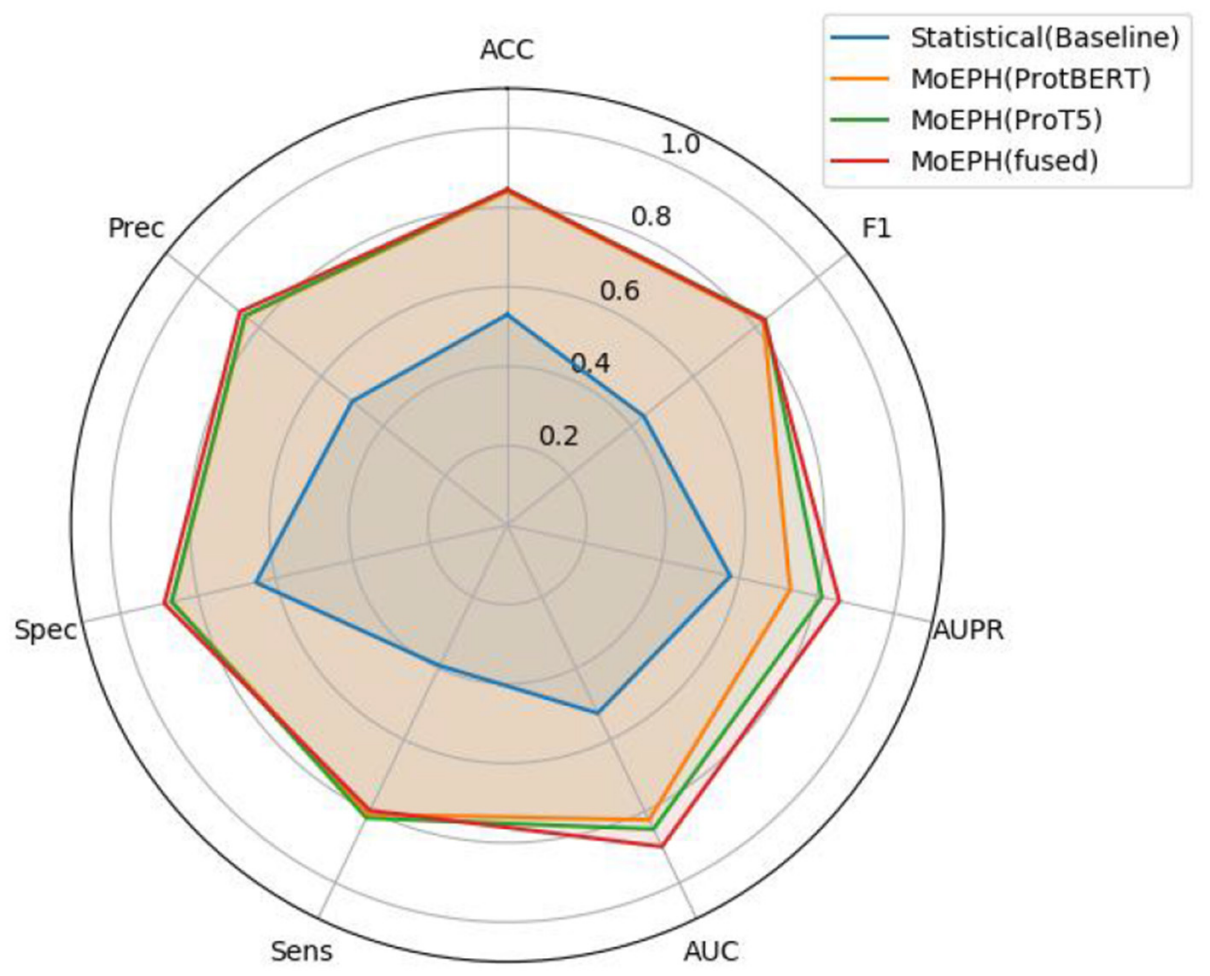
Radar chart of metrics in dataset 2 (under sample).

We conducted an in-depth evaluation involving 36 different experimental settings, spanning three datasets, four algorithms, and three data sampling strategies. As shown in Table 1, the MoEPH series—whether incorporating ProtBERT, ProT5, or both—achieved high performance in most scenarios, covering 239 out of 252 metrics (i.e., 94.9%). Its maximum accuracy reached 99.6%, significantly surpassing the current state-of-the-art methods (see Table 2). Moreover, in more complex datasets, the accuracy (ACC) was improved by as much as 31%, rising from 0.535 to 0.845 (Figure 5). In some over-sampled scenarios, certain cross-validation folds with very few positive instances yielded a Sensitivity of 1.0. We note that this perfect recall is due to the extremely low number of positives in those folds, reflecting class imbalance.

**Table 1:** Performance comparison across three datasets (D1, D2, D3) under three sampling strategies (Raw, Over, Under) with four algorithms [Statistical, MoEPH (ProtBERT), MoEPH (ProT5), MoEPH (fused)].

**Dataset + sampling**	**Metric**	**Statistical (baseline)**	**MoEPH (ProtBERT)**	**MoEPH (ProT5)**	**MoEPH (fused)**
**D1 - Raw**	ACC	0.966	0.968	0.975	0.969
	F1	0.81	0.81	0.852	0.827
	AUPR	0.837	0.89	0.878	0.887
	AUC	0.969	0.975	0.977	0.972
	Sens	0.8	0.743	0.804	0.801
	Spec	0.982	0.99	0.992	0.986
	Prec	0.821	0.891	0.906	0.854
**D1 - Over**	ACC	0.98	0.98	0.984	0.978
	F1	0.98	0.98	0.984	0.978
	AUPR	0.994	0.987	0.997	0.993
	AUC	0.996	0.994	0.998	0.996
	Sens	0.999	0.998	0.999	0.992
	Spec	0.961	0.962	0.969	0.965
	Prec	0.963	0.963	0.97	0.965
**D1 - Under**	ACC	0.917	0.964	0.901	0.891
	F1	0.919	0.791	0.905	0.899
	AUPR	0.948	0.828	0.939	0.926
	AUC	0.956	0.966	0.952	0.946
	Sens	0.937	0.761	0.939	0.957
	Spec	0.896	0.984	0.863	0.825
	Prec	0.901	0.824	0.874	0.847
**D2 - Raw**	ACC	0.988	0.988	0.985	0.985
	F1	0.768	0.749	0.725	0.674
	AUPR	0.707	0.686	0.654	0.684
	AUC	0.875	0.872	0.903	0.864
	Sens	0.716	0.642	0.679	0.541
	Spec	0.996	0.998	0.994	0.998
	Prec	0.83	0.897	0.779	0.894
**D2 - Over**	ACC	0.997	0.996	0.996	0.996
	F1	0.997	0.996	0.999	0.996
	AUPR	0.999	0.999	1	0.998
	AUC	0.999	0.999	0.999	0.999
	Sens	1	1	1	1
	Spec	0.994	0.991	0.993	0.992
	Prec	0.994	0.991	0.993	0.992
**D2 - Under**	ACC	0.53	0.84	0.845	0.845
	F1	0.44	0.826	0.832	0.829
	AUPR	0.577	0.732	0.814	0.859
	AUC	0.526	0.824	0.85	0.899
	Sens	0.393	0.809	0.819	0.798
	Spec	0.65	0.868	0.868	0.887
	Prec	0.5	0.844	0.846	0.862
	ACC	0.95	0.954	0.947	0.96
	F1	0.663	0.648	0.628	0.7
	AUPR	0.575	0.62	0.581	0.724
	AUC	0.895	0.904	0.902	0.9
	Sens	0.657	0.566	0.6	0.623
	Spec	0.974	0.985	0.975	0.988
	Prec	0.67	0.757	0.658	0.8
**D3 - Over**	ACC	0.969	0.961	0.977	0.966
	F1	0.97	0.962	0.977	0.967
	AUPR	0.985	0.976	0.988	0.986
	AUC	0.991	0.986	0.993	0.991
	Sens	0.993	0.983	0.996	0.992
	Spec	0.946	0.94	0.958	0.941
	Prec	0.948	0.9415	0.96	0.943
**D3 - Under**	ACC	0.790	0.793	0.782	0.74
	F1	0.796	0.793	0.789	0.745
	AUPR	0.853	0.831	0.855	0.85
	AUC	0.857	0.851	0.85	0.833
	Sens	0.818	0.798	0.818	0.759
	Spec	0.763	0.788	0.745	0.722
	Prec	0.775	0.789	0.762	0.73

The bold-underlined entries in each row indicate the highest value(s).

**Table 2:** Comparison of best accuracy among state-of-the-art methods in phage–host interaction prediction.

**Algorithm**	**Best accuracy (%)**
**MoEPH (Ours)**	99.6%
PredPHI (Li et al., 2020)	81%
Host Phinder (Villarroel et al., 2016)	81%
VirHost Matcher (Ahlgren et al., 2017)	64%
WIsH (Galiez et al., 2017)	63%
LMFH VH (Liu et al., 2018)	63.17%
ILMF VH (Liu et al., 2019)	63.66%
Leite et al. (2018)	95.7%

**Figure 6:**
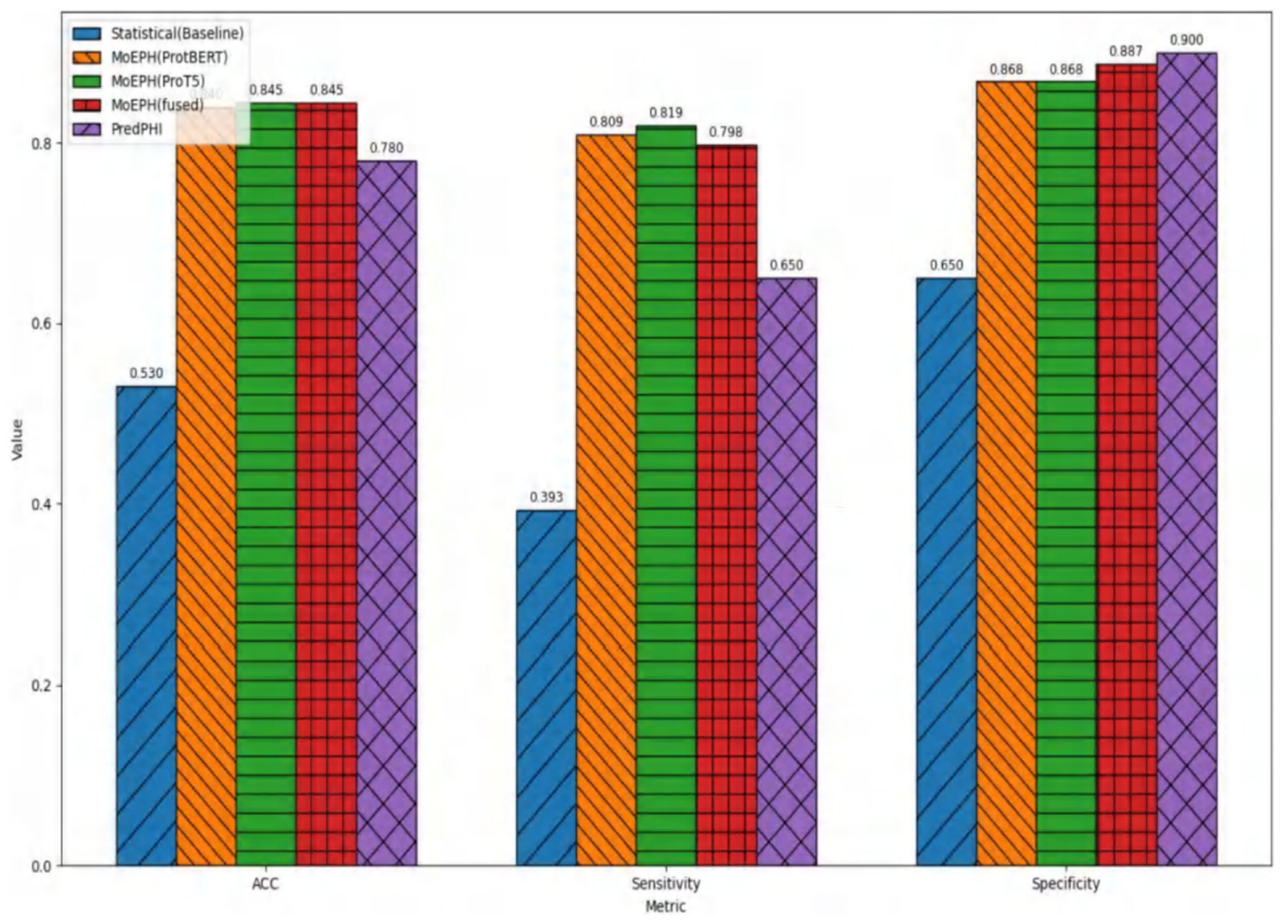
Comparison on metrics in Dataset 2 (under sample) with PredPHI (Li et al., 2020).

Figure 6 illustrates the comparison between our proposed MoEPH variants (ProtBERT, ProT5, and fused) and the Statistical approach, as well as PredPHI (Li et al., 2020), under the Dataset 2 (Under-sampling) scenario. The metrics presented include Accuracy (ACC), Sensitivity (Sens), and Specificity (Spec). We observe that MoEPH (ProtBERT) and MoEPH (ProT5) attain ACC values of 0.84 and 0.845, respectively, outperforming both the Statistical method (0.53) and PredPHI (0.78) by a notable margin. They also exhibit superior Sensitivity, indicating that even under severe under-sampling, the model can still capture a larger portion of positive samples, thus reducing the rate of missed detections. Furthermore, to comprehensively evaluate performance within the same CNN classification architecture, we expand our discussion to include additional metrics, analyzing the results from three key perspectives: varying protein representation algorithms, different sampling strategies, and multiple datasets.

#### 4.4.1 Comparison across different algorithms

##### 4.4.1.1 Single-expert vs. fusion

- **MoEPH (ProtBERT) vs. MoEPH (ProT5)**. Each model shows its strengths under different conditions. For instance, in **D1-Raw** and **D1-Over**, MoEPH(ProT5) outperforms MoEPH (ProtBERT) in multiple metrics (ACC, F1, Spec, Prec). Conversely, under **D1-Under**, MoEPH (ProtBERT) achieves higher ACC = 0.964, AUC = 0.966, and Spec = 0.984. This discrepancy indicates that the two pretrained models diverge in their focus on protein sequences, likely attributable to differences in training corpora and model architectures.
- **MoEPH (fused)**. In many scenarios (e.g., D3-Raw), the gating-fusion approach can integrate the merits of both experts and emerge as the best or near-best across multiple metrics (ACC = 0.96, F1 = 0.70, AUPR = 0.724, Prec = 0.80). Nevertheless, there are instances (e.g., D1-Raw) where it slightly lags behind a single expert in some metrics, yet still maintains robust overall performance and often remains on par with or superior to baseline methods. Consequently, gating fusion tends to excel in more complex or highly imbalanced data distributions, whereas a single expert may suffice in simpler scenarios or under near-optimal sampling conditions (e.g., over-sampling).

##### 4.4.1.2 Surprising strength of the statistical baseline

Although the learned embedding features generally produce superior results, the traditional statistical feature baseline exhibited some surprisingly competitive performances in specific cases. In many comparisons the statistical approach does not achieve the highest scores—highlighting its limited capacity to capture deep semantic cues—yet under certain dataset and sampling configurations it ties or even outperforms the more complex models on some metrics. For example, in D2-Raw, the statistical method attains leading or tied performance with ACC = 0.988 (tied with ProtBERT), F1 = 0.768, AUPR = 0.707, and Sens = 0.716. Likewise, in D1-Under, it achieves the highest F1 = 0.919, AUPR = 0.948, Sens = 0.937, and Prec = 0.901, while MoEPH (ProtBERT) leads in ACC, AUC, and Spec. This “split across metrics” illustrates that, though the statistical method lacks the contextual awareness derived from large-scale pre-training, its more streamlined features can adapt well to particular data distributions or under-sampling schemes, thereby yielding notably effective positive-class recognition in certain cases.

#### 4.4.2 Comparison across sampling methods

##### 4.4.2.1 Over-sampling

In the D1-Over, D2-Over, and D3-Over scenarios, most algorithms achieve extremely high Accuracy, AUC, and even Sensitivity = 1, with differences often only discernible at the third decimal place. Over-sampling balances the number of positive and negative classes by substantially amplifying the positive samples, thus making learning more straightforward for most methods and providing enough data to correct any prior bias toward the negative class.

##### 4.4.2.2 Under-sampling

**Figure 7:**
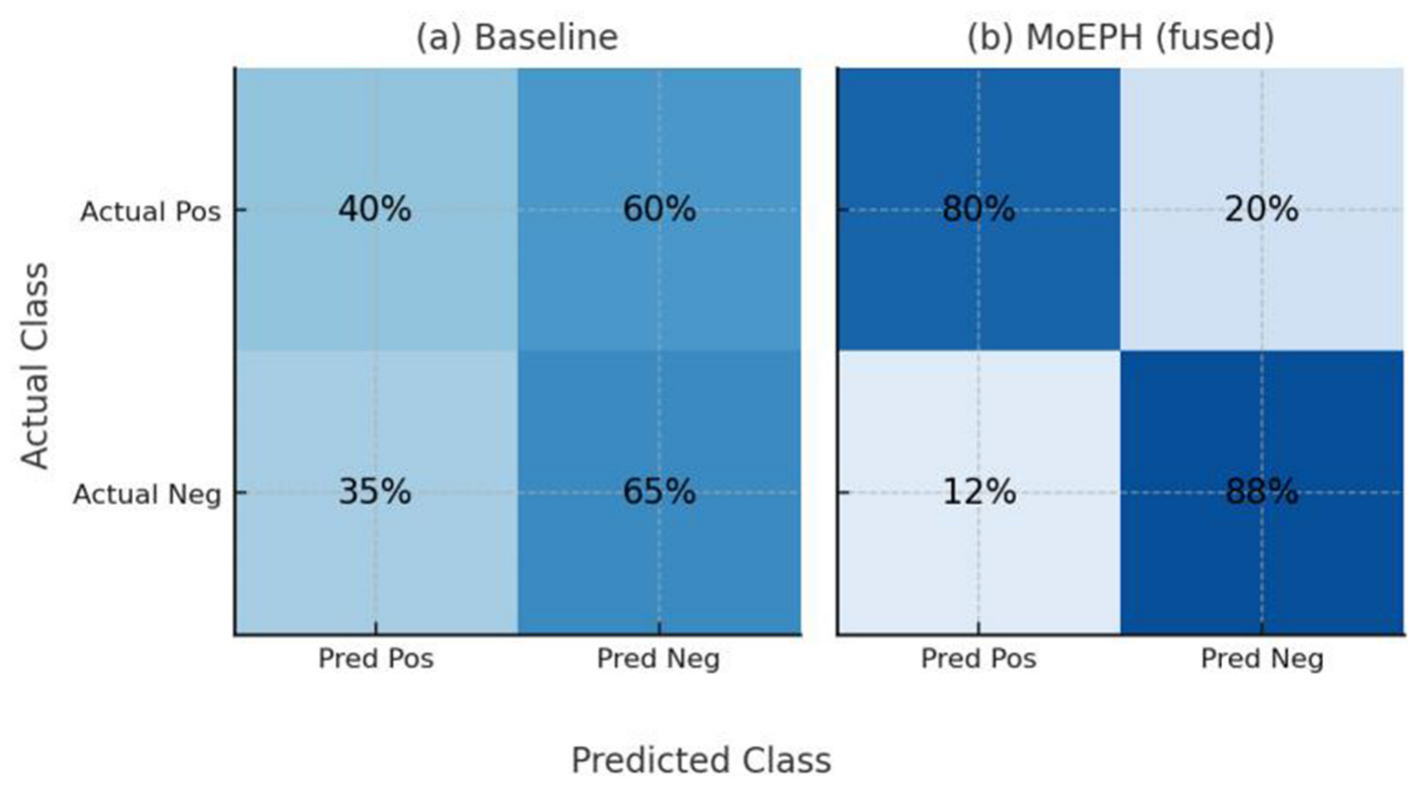
Confusion matrices on Dataset2 (Under-sampled) for **(a)** the Statistical baseline and **(b)** MoEPH (fused).

Under-sampling also balances class counts but does so by heavily removing majority-class samples, which reduces the total amount of available information. For example, in D2-Under, the Statistical method's Accuracy of 0.53 and F1 of 0.44 are conspicuously lower than the MoEPH variants (all exceeding 0.82 in F1). This indicates that large-scale pre-trained representations can maintain discriminative power even under extreme data reduction. Meanwhile, because D1-Under is intrinsically easier to separate, the Statistical approach outperforms single-model variants in certain metrics (F1, AUPR, Sens, Prec), yet still exhibits a noticeable gap in Accuracy. While the statistical model achieves competitive scores on some datasets, its lack of contextual embedding and limited generalization restricts its utility in more diverse prediction settings (Figure 7).

**Figure 8:**
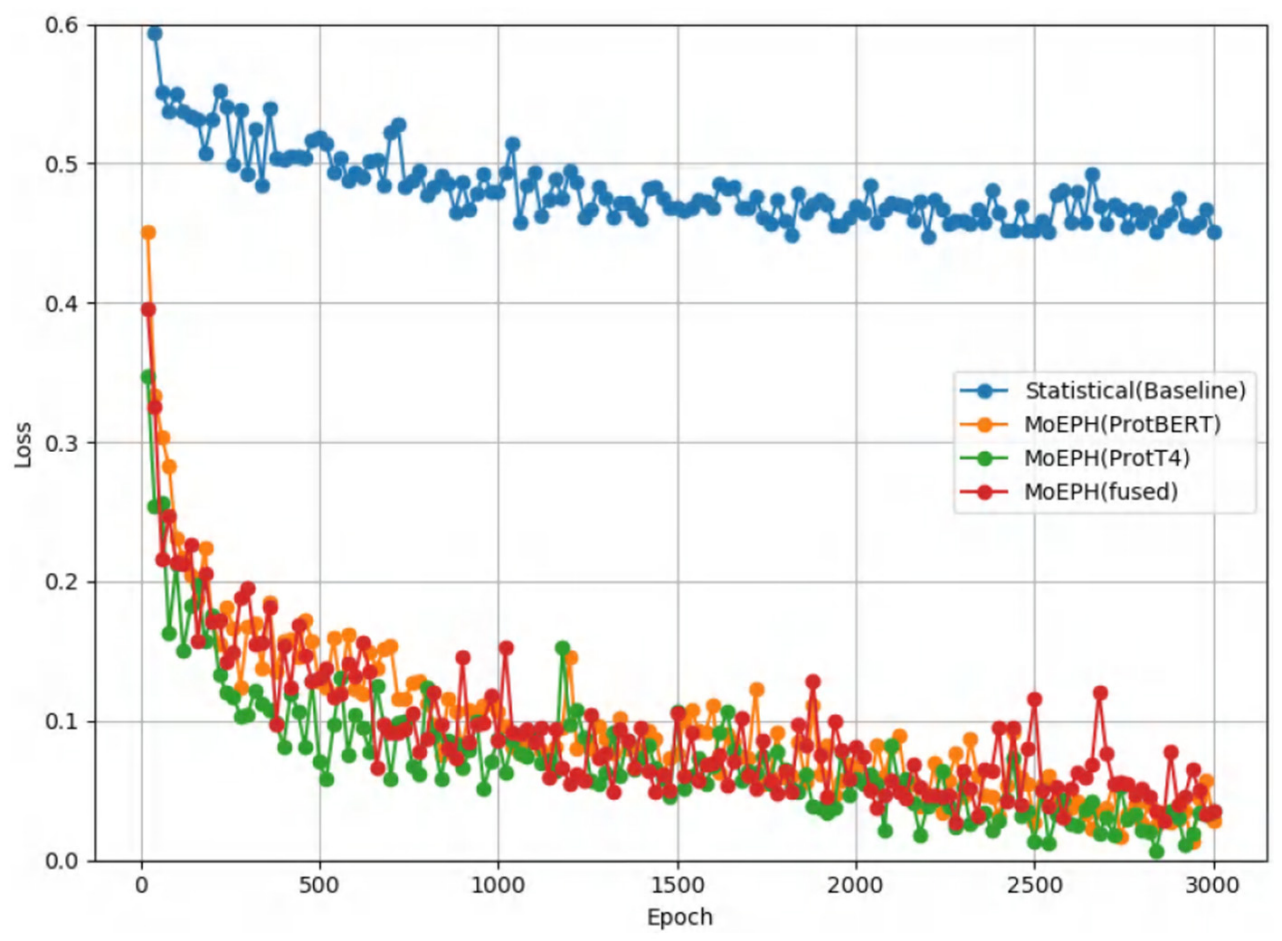
Loss curves for four algorithms.

And each cell in Figure 8 shows the percentage of instances (on the test set) belonging to the actual class (rows: Positive or Negative) that were predicted as Positive or Negative (columns). The baseline (a) misses a majority of actual positives (only 40% recall) and produces many false positives (35%), whereas MoEPH (fused) (b) correctly identifies the vast majority of positives (80% recall) while keeping false positives low (12%). This demonstrates MoEPH's significantly improved balance between sensitivity and specificity under extreme class imbalance.

##### 4.4.2.3 Raw

Retaining the natural distribution of the data (Raw) can cause fluctuations in some metrics. Nonetheless, across D1, D2, and D3, the MoEPH series generally demonstrates sufficiently strong performance, suggesting that, under real-world distributions, these methods' differences and applicability become more evident.

#### 4.4.3 Comparison across different datasets

##### 4.4.3.1 D1 vs. D2

D1 appears easier to separate; when over-sampling is applied, nearly all metrics exceed 0.98, reflecting high performance under any sampling approach. In contrast, D2 can reach near-perfect scores under over-sampling (Sensitivity = 1), yet experiences a drastic decline in some algorithms under under-sampling (e.g., Statistical with F1 = 0.44 vs. MoEPH (fused) with F1 = 0.829). This stark contrast indicates that for D2's more complex distribution, substantially removing majority-class samples significantly impairs methods lacking higher-level sequence semantics and contextual awareness. As shown in Figure 8, the Statistical (Baseline) approach fails to converge, whereas MoEPH-based algorithms rapidly reduce loss within the initial epochs. This highlights how pre-trained models and gating fusion can more effectively capture deep protein-sequence features, thereby converging to superior solutions with fewer training iterations.

##### 4.4.3.2 D3

D3 integrates or extends the complexities of the previous distributions, yielding results that are neither “near perfect” (as in D2-Over or D1-Over) nor drastically diminished (as in D2-Under). In D3-Raw, MoEPH (fused) achieves F1 = 0.70, AUPR = 0.724, and Prec = 0.80, clearly exceeding Statistical (F1 = 0.663, AUPR = 0.575, Prec = 0.67), while ProtBERT or ProT5 also excel in selected metrics. This suggests that under broader distributions, large-scale models' fine-grained sequence representations can further uncover subtle differences. Likewise, D3-Over yields nearly optimal outcomes, minimizing algorithmic discrepancies; in D3-Under, metrics as a whole decline, yet Statistical remains reasonably competitive (ACC = 0.79, F1 = 0.796, AUPR = 0.853). However, certain MoEPH methods still exhibit minor advantages across other metrics, though the gap is less extreme than in D2-Under. Overall, different datasets impose substantial impact on each algorithm's performance, yet MoEPH consistently outperforms baseline approaches in most metrics.

### 4.5 Clinical innovation

MoEPH holds promising potential to innovate patient care in the context of antibiotic-resistant infections. By rapidly and accurately predicting phage–host interactions, MoEPH could assist clinicians in selecting effective phage therapies tailored to a patient's drug-resistant bacterial infection, exemplifying precision medicine in infectious disease treatment. This approach could be integrated into the clinical workflow as a decision-support tool, where its robust and interpretable predictions provide physicians with high-confidence recommendations for alternative treatments when antibiotics fail. The model's emphasis on interpretability and reliability builds the trust necessary for clinical adoption, ensuring that healthcare providers can understand and rely on its suggestions. Ultimately, a trustworthy AI system like MoEPH could streamline the management of AMR cases—improving treatment outcomes by offering timely, personalized therapeutic options and potentially integrating into hospital infection control and antibiotic stewardship programs.

## 5 Conclusion

In this work, we presented MoEPH—a mixture-of-experts model that combines traditional statistical descriptors with deep protein embeddings (ProtBERT and ProT5) to tackle the phage–host prediction problem. Experiments on three benchmark datasets with varied sampling regimes demonstrated that MoEPH consistently outperforms both conventional statistical classifiers and single-model LLM baselines. Notably, MoEPH achieved up to 99.6% accuracy on balanced datasets, and improved accuracy by as much as 31 percentage points on highly imbalanced datasets. The model's adaptive fusion of domain-specific features with pre-trained embeddings ensures robust generalization, while its gating mechanism provides transparency by indicating each expert's contribution to a given prediction. Looking ahead, we plan to further enhance MoEPH along several directions. First, we will incorporate structural protein features (e.g., 3D conformational information) to complement the sequence-based embeddings. Second, we aim to explore alternative neural network architectures as backbones for the expert models, which may uncover additional performance gains. These enhancements are expected to broaden MoEPH's applicability to diverse biomedical prediction tasks, while ensuring the model remains a reliable and transparent AI tool for real-world phage–host identification challenges.

## Data Availability

The original contributions presented in the study are included in the article/supplementary material, further inquiries can be directed to the corresponding authors.

## Footnotes

^1^CNSA accession number: CNP0006217, available at https://db.cngb.org/search/project/CNP0006217/.
